# The zebrafish embryo as an *in vivo* model for screening nanoparticle-formulated lipophilic anti-tuberculosis compounds

**DOI:** 10.1242/dmm.049147

**Published:** 2022-01-26

**Authors:** Nils-Jørgen Knudsen Dal, Martin Speth, Kerstin Johann, Matthias Barz, Claire Beauvineau, Jens Wohlmann, Federico Fenaroli, Brigitte Gicquel, Gareth Griffiths, Noelia Alonso-Rodriguez

**Affiliations:** 1 Department Biosciences, Faculty of Mathematics and Natural Sciences, University of Oslo, 0371 Oslo, Norway; 2 Department of Chemistry, Johannes Gutenberg University Mainz, 55128 Mainz, Germany; 3 Division of BioTherapeutics, Leiden Academic Center for Drug Research (LACDR), Leiden University, 2333 Leiden, The Netherlands; 4 Chemical Library Institut Curie/CNRS, CNRS UMR9187, INSERM U1196 and CNRS UMR3666, INSERM U1193, Université Paris-Saclay, F-91405 Orsay, France; 5 Unité de Génétique Mycobactérienne, Dep Génomes and Génétique, Institute Pasteur, 75015 Paris, France; 6 Department of Tuberculosis Control and Prevention, Shenzhen Nanshan Center for Chronic Disease Control, 518054 Shenzhen, China

**Keywords:** Zebrafish tuberculosis model, Anti-tuberculosis drugs, Nanoparticles, *In vivo* toxicity, *In vivo* efficacy

## Abstract

With the increasing emergence of drug-resistant *Mycobacterium tuberculosis* strains, new and effective antibiotics against tuberculosis (TB) are urgently needed. However, the high frequency of poorly water-soluble compounds among hits in high-throughput drug screening campaigns is a major obstacle in drug discovery. Moreover, *in vivo* testing using conventional animal TB models, such as mice, is time consuming and costly, and represents a major bottleneck in lead compound discovery and development. Here, we report the use of the zebrafish embryo TB model for evaluating the *in vivo* toxicity and efficacy of five poorly water-soluble nitronaphthofuran derivatives, which were recently identified as possessing anti-TB activity *in vitro*. To aid solubilization, compounds were formulated in biocompatible polymeric micelles (PMs). Three of the five PM-formulated nitronaphthofuran derivatives showed low toxicity *in vivo*, significantly reduced bacterial burden and improved survival in infected zebrafish embryos. We propose the zebrafish embryo TB-model as a quick and sensitive tool for evaluating the *in vivo* toxicity and efficacy of new anti-TB compounds during early stages of drug development. Thus, this model is well suited for pinpointing promising compounds for further development.

## Abstract (precis)

**Summary:** The zebrafish embryo is a quick, sensitive and powerful tool for evaluating the *in vivo* toxicity and efficacy of newly identified lipophilic anti-tuberculosis compounds formulated in nanoparticles.

## INTRODUCTION

Tuberculosis (TB), caused by the bacterium *Mycobacterium tuberculosis* (Mtb), is one of the deadliest infectious diseases worldwide. Annually, ten million people fall ill with TB, of whom ∼1.4 million die (World Health Organization, 2020). Reducing the total burden of TB is difficult due to the extensive reservoir of latently infected individuals who are at risk of developing active disease in their lifetime (Houben and Dodd, 2016). The alarming increase in cases of drug-resistant TB in recent years highlights the urgency for new effective anti TB-drugs or regimens that will shorten the duration of treatment and improve cure rates (Lange et al., 2018; World Health Organization, 2020).

In recent years, the use of phenotypic high throughput screening (HTS) of large-scale compound libraries against whole-cell Mtb or surrogate mycobacteria, such as *Mycobacterium bovis* BCG, has proven to be a powerful strategy in the quest to identify new anti-TB drug candidates. Phenotypic HTS has resulted in the discovery of recently approved anti-TB drugs, such as bedaquiline, pretomanid and delamanid, as well as other anti-TB drug candidates currently in clinical trials (Grzelak et al., 2019). However, the phenotypic HTS approach has been shown to be inherently biased towards identifying lipophilic, and thus less water-soluble, molecules (Goldman, 2013). Indeed, lipophilicity is positively correlated with anti-mycobacterial activity, likely due to the fact that the thick lipid-rich and waxy cell wall structure of Mtb constitutes a close to impenetrable barrier for hydrophilic molecules (Machado et al., 2018). However, high lipophilicity and low water solubility is considered to be a ‘red flag’ in medicinal chemistry due to the potential negative effects on absorption, distribution, metabolism and elimination, which are often associated with toxic properties of candidate compounds (Lipinski, 2000; Tarcsay and Keseru, 2013; Waring, 2010). Such compounds are either rejected from the drug discovery process at early stages or are subjected to chemical modifications, with the aim of reducing their lipophilic nature and increasing their water solubility, thus making them easier to administer and improving their bioavailability (Di et al., 2012). However, alteration of the drug structure presents the risk of reducing the anti-mycobacterial activity (Kondreddi et al., 2013; Manjunatha and Smith, 2015).

In recent years, nanoparticle technology has emerged as a promising alternative approach to improve unfavorable pharmacokinetic characteristics and the low bioavailability of poorly water-soluble compounds (Kirtane et al., 2021). By virtue of their ability to encapsulate drugs in their matrix or core, nanoparticle-based formulations increase the bioavailability of lipophilic drugs and reduce drug-related toxicity (Kirtane et al., 2021; Melariri et al., 2015; Vibe et al., 2016). Importantly, nanoparticles can be designed to release their drug load in a sustained manner, thereby maintaining systemic levels above the minimum effective concentration over prolonged periods and, as a consequence, increasing the efficacy of drugs compared to the application of the free drugs alone (Griffiths et al., 2010; Sharma et al., 2004).

In many cases, overcoming poor solubility is a major challenge when progressing lipophilic drug candidates to the preclinical test stage and, in particular, to efficacy studies in animal models of TB. The *in vivo* efficacy of new anti-TB drug candidates is predominately assessed in conventional mouse or guinea pig models of TB. These *in vivo* studies are generally costly, time consuming and require advanced biosafety level-3 research and animal facilities, thereby representing a considerable bottleneck in the drug discovery process (Andreu et al., 2013; Balganesh et al., 2004). In recent years, the zebrafish embryo (*Danio rerio*) has been established as a powerful alternative *in vivo* TB model. Infection of zebrafish embryos with the natural fish pathogen *Mycobacterium marinum* (Mm), a genetically close relative of Mtb, results in disease that recapitulates many key features of the early stages of Mtb infection. Taking advantage of the transparent nature of the zebrafish embryo, major new insights have been achieved into the interaction of mycobacterial pathogens with host cells during early phases of infection (Davis et al., 2002; Johansen et al., 2018; Tobin and Ramakrishnan, 2008; Volkman et al., 2004). Moreover, the zebrafish TB model has been used to evaluate the *in vivo* efficacy of anti-TB drug candidates (Carvalho et al., 2011; Dalton et al., 2017; Makarov et al., 2014; Ordas et al., 2015; Takaki et al., 2012). The zebrafish embryo is also a well-established model organism in toxicology research, and has been widely used to evaluate the *in vivo* toxicity of candidate drugs against a variety of diseases, such as epilepsy, cancer and bacterial infections (Cassar et al., 2020). Embryos can be conveniently kept in multiwell dishes and have been successfully used in numerous high-throughput toxicology screening studies, such as those focusing on cardiotoxicity, hepatotoxicity and adverse behavioral effects (Cornet et al., 2017; Dyballa et al., 2019; Zhang et al., 2017).

In previous work, our group has used the zebrafish embryo model to characterize the toxicity and the efficacy of anti-TB drug-loaded nanoparticles against Mm *in vivo* (Fenaroli et al., 2014; Vibe et al., 2016). This system also allowed us to monitor important parameters of nanoparticles in live animals, such as their persistence and stability in the blood circulation, interaction with host immune cells, biodistribution and dynamics of accumulation at the infection sites, the granulomas. Importantly, a number of key findings from the zebrafish model could be replicated in Mtb-infected mice; in particular, nanoparticles in both model systems exhibited similar accumulation in granulomas and comparable stability in the bloodstream (Dal et al., 2020; Fenaroli et al., 2018).

In this study, we demonstrate the use of the zebrafish embryo to evaluate both *in vivo* toxicity and *in vivo* anti-mycobacterial efficacy of nitronaphthofuran (nNF) compounds that were previously identified to be active against Mtb in a phenotypic HTS at the Institute Pasteur (Paris, France, data not published). The five most promising nNF-derivatives showing potent anti-TB *in vitro* activity (MIC_90_<8 µg/ml) were selected for this study. As the water solubility of the selected derivatives is low (<12 µg/ml), we opted for formulating the compounds in biocompatible polymeric micelles (PMs), primarily to aid their solubilization and to be able to administer therapeutic doses in the zebrafish embryo model without the use of toxic solvents. Importantly, we delivered the candidate drugs directly into the blood circulation of zebrafish embryos by intravenous injection, thereby circumventing absorption issues associated with delivery by bath immersion, as used in most previously published studies. By using a set of complementary *in vivo* assays we were then able to pinpoint the most promising nNF candidates for further development in the drug discovery process.

## RESULTS

**Fig. 1.**
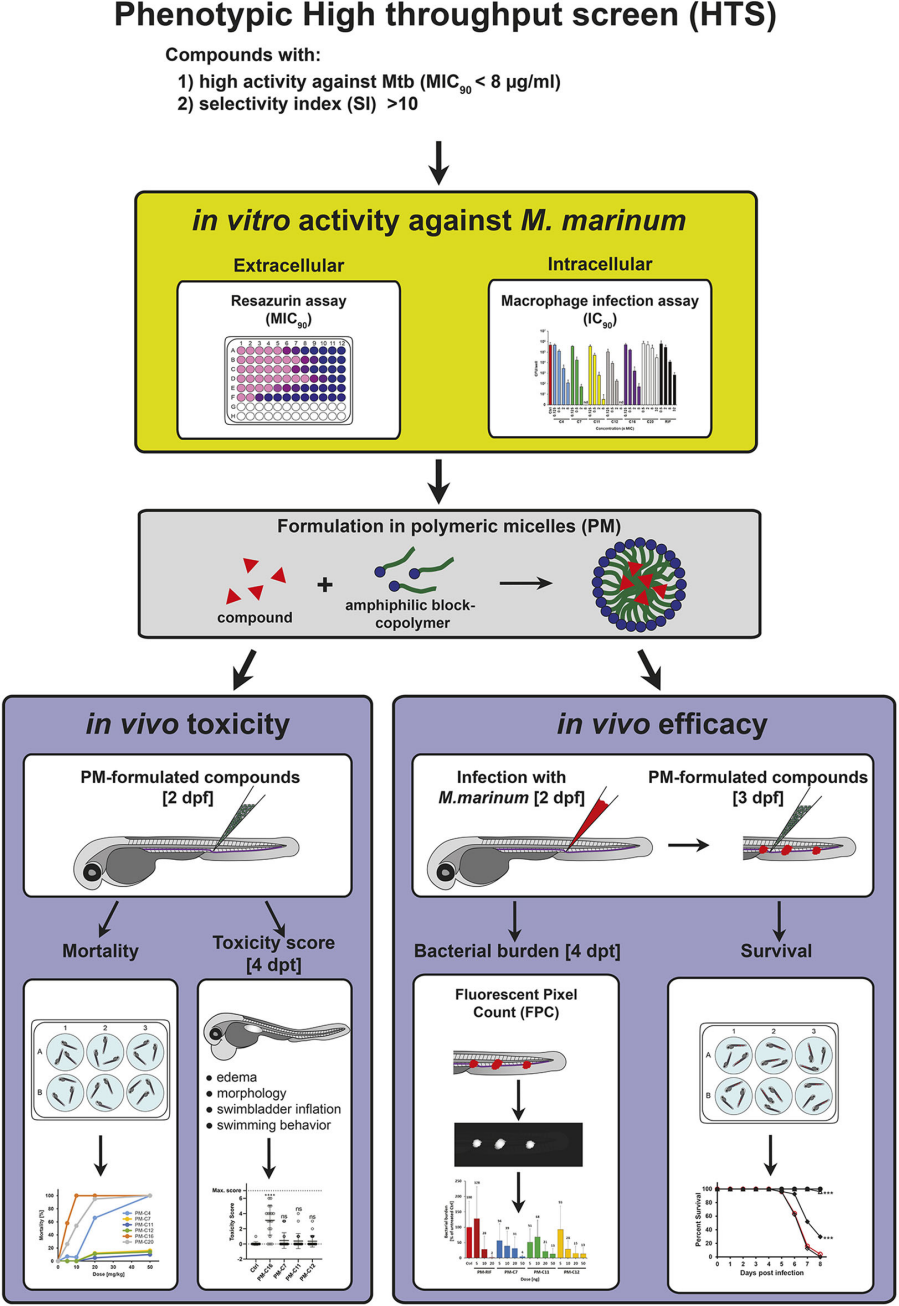
**Schematic summary of the strategy used to evaluate the *in vivo* toxicity and anti-mycobacterial efficacy of HTS-identified lipophilic compounds using the zebrafish embryo model.** Candidate compounds with significant anti-mycobacterial activity were identified in a phenotypic HTS campaign using chemical libraries and selected for further evaluation based on their *in vitro* activity against Mtb (MIC_90_), and their selectivity index (SI). Prior to *in vivo* testing in the zebrafish TB model using the fish pathogen Mm, candidate compounds were tested for their *in vitro* activity against extracellular Mm using a resazurin assay and against intracellular bacteria in mouse macrophages using a CFU assay. Then, candidate compounds were formulated in PMs, primarily to enhance solubility and to enable intravenous injection in the zebrafish embryo. The *in vivo* toxicity of PM-formulated compounds was first evaluated by mortality analysis after a single injection of different doses at 8 days post treatment (dpt), and compounds with significant toxicity were excluded from further analysis. PM-formulated compounds showing low mortality were then characterized in more detail by assigning a toxicity score to individual embryos at 4 dpt based on different morphological and physiological indicators of toxicity, as outlined in Materials and Methods. For the evaluation of *in vivo* efficacy, zebrafish embryos were infected with Mm and treated with different doses of PM-formulated compounds. Therapeutic efficacy was then assessed by bacterial burden (FPC) at 4 dpt and by survival analysis. dpf, days post fertilization.

In this study we made use of the zebrafish embryo model to evaluate the *in vivo* toxicity and efficacy of new anti-TB compounds, which were initially identified in a previous phenotypic high-throughput screening at the Institute Pasteur (Paris, France). The strategy and methods we used are summarized in Fig. 1 and included *in vitro* cell culture based assays, as well as several complementary assays for *in vivo* toxicity and efficacy in the zebrafish embryo.

### *In vitro* activity of nNF – derivatives against extracellular and intracellular *M. marinum*

**Fig. 2.**
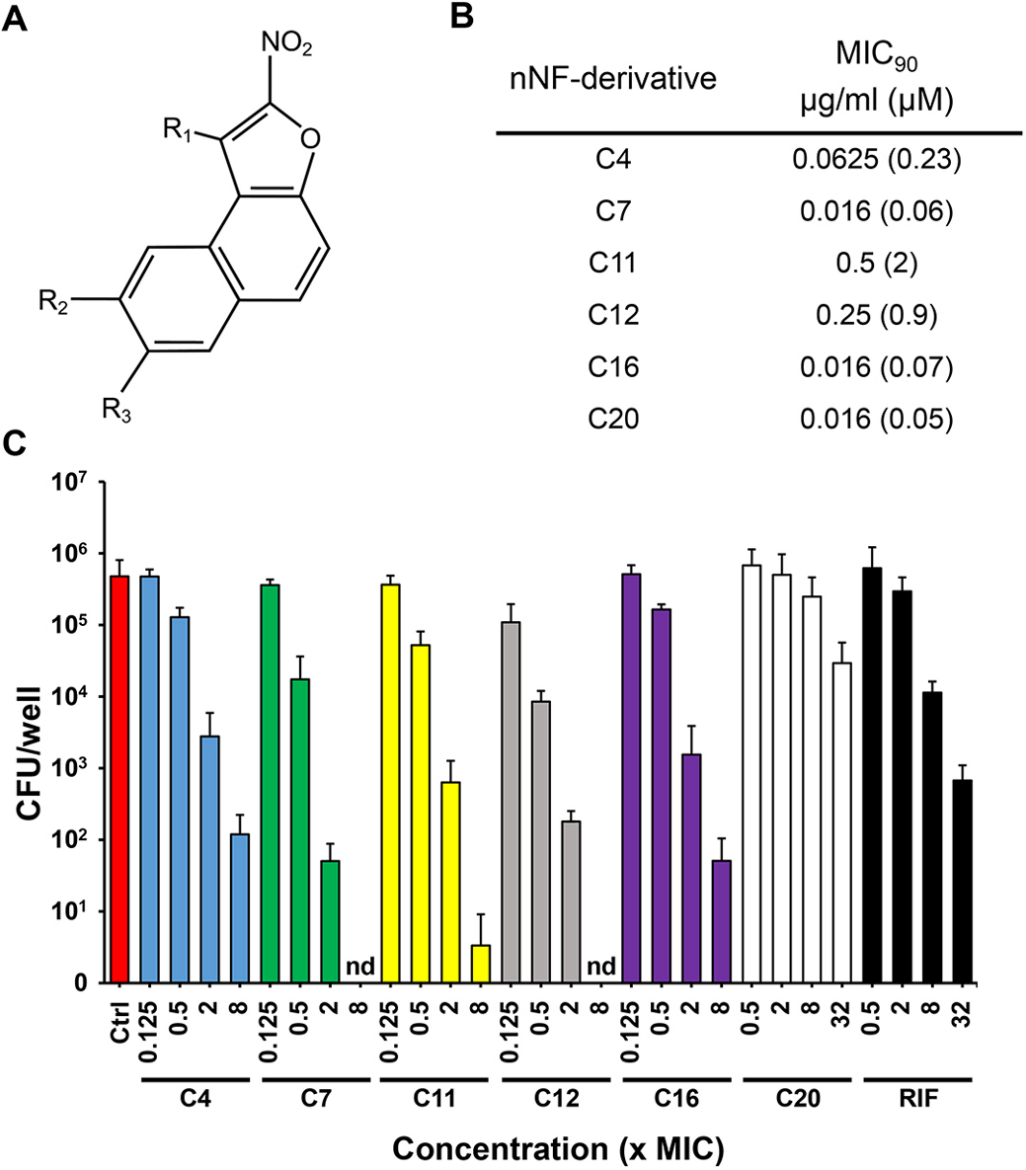
***In vitro* anti-mycobacterial activity of nNF derivatives against extracellular and intracellular Mm.** (A) The nitroaromatic backbone structure of the nNF compounds tested in this study. (B) Activity of nNF derivatives against extracellular Mm determined by MIC_90_. (C) Anti-mycobacterial activity of nNF derivatives and RIF against intracellular Mm in murine BMDMs. BMDM cultures were infected with Mm at 32.5°C at a MOI of 0.1 and treated with nNF derivatives at different concentrations based on their MIC_90_. Intracellular mycobacterial burden was determined by CFU plating at 4 days post infection. Data represent the mean±s.d. of three independent experiments. nd, not detected.

As we aimed to evaluate the *in vivo* efficacy of these compounds in the zebrafish TB model using Mm, we first assessed their activity against extracellular Mm in a minimum inhibitory concentration (MIC) assay. We tested five novel nNF derivatives, the molecular structures of which are characterized by a furan ring bearing a nitro group (Fig. 2A). All of them were found to be potent against extracellular Mm, with MIC_90_ values ranging from 0.016 to 0.5 µg/ml (Fig. 2B). To test their activity against intracellular Mm, we treated Mm-infected mouse bone marrow-derived macrophages (BMDMs) with the nNF compounds and the established anti-TB drug rifampicin (RIF), and enumerated intracellular bacteria by counting colony-forming units (CFUs). At the tested concentration range – from 0.125× to 32× the corresponding MIC_90_ – none of the nNF compounds showed cytotoxic effects in BMDM cultures (Fig. S1). With the exception of C20 and RIF, all compounds showed significant efficacy against intracellular Mm with a 2-4 log reduction in bacterial burden at 2× the MIC_90_ (Fig. 2C). Higher concentrations (8× the MIC_90_) resulted in almost complete eradication of intracellular Mm, with no detectable bacterial colonies in the case of C7 and C12. Compound C20, despite being highly effective against extracellular Mm, showed relatively low efficacy against intracellular Mm within the studied concentration range. In general, compounds C4, C7, C11 and C12 showed comparable activity against intracellular and extracellular Mm, with an IC_90_ (90% inhibitory concentration) against intracellular bacteria lower or equivalent to their respective MIC_90_ (Table S1). In contrast, C20 and RIF showed significantly lower activity against intracellular Mm, with a 26× and 9× higher IC_90_ than their MIC_90_, respectively.

### Formulation of nNF-derivatives and RIF in PMs

The low water solubility of the selected nNF-derivatives (predicted to be between 0.6 and 12 µg/ml) did not allow us to inject therapeutic doses without the use of toxic concentrations of solvents, such as DMSO (data not shown). Therefore, in order to evaluate them in the zebrafish embryo, we opted to encapsulate the nNF-derivatives in biocompatible and biodegradable polypept(o)ide block copolymer [pGlu(OBn)-block-pSar] using dual asymmetric centrifugation. This produced drug-encapsulated PMs with a size (diameter) between 84 and 149 nm, a narrow size distribution (polydispersity index≤0.15) and a slightly negative surface charge ranging from −3.7 mV to −2.5 mV, as measured by zeta-potential (Table S2). By employing this strategy, we were able to significantly increase compound solubility and therefore inject zebrafish embryos with the candidate compounds in a therapeutic dose range, avoiding the use of toxic solvents.

### *In vivo* toxicity of PM-formulated nNF derivatives in the zebrafish embryo

**Fig. 3.**
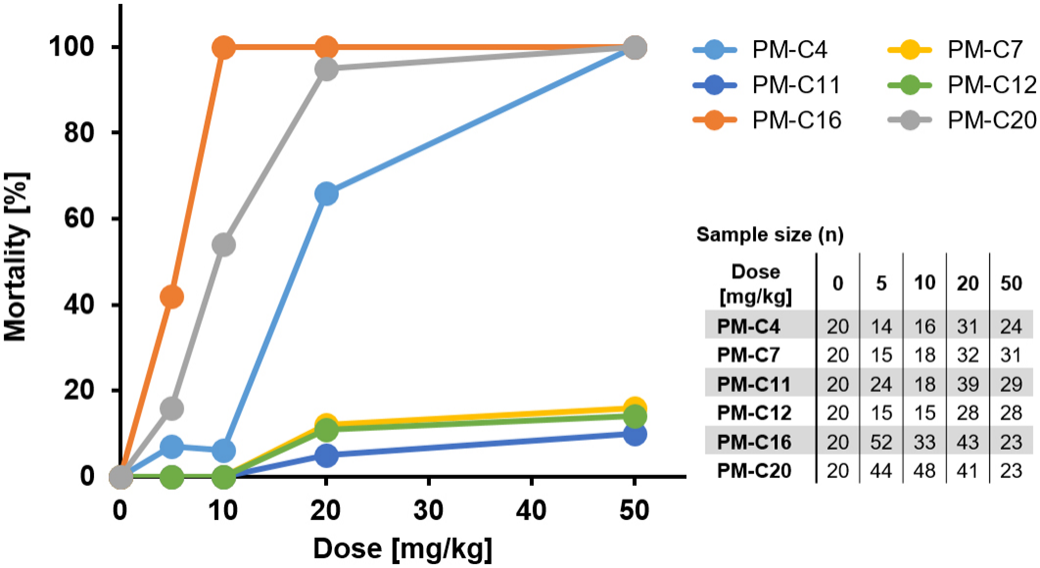
**Evaluation of the *in vivo* toxicity of PM-formulated nNF derivatives in zebrafish embryos by mortality analysis.** Zebrafish embryos were treated with different doses of PM-formulated nNF derivatives by intravenous injection, and mortality was recorded at 8 dpt. Results are pooled from at least three independent experiments.

The *in vivo* toxicity of the PM-formulated compounds was tested in zebrafish embryos using a mortality assay after the injection of different doses into the posterior caudal vein (PCV). As expected, PM-C16 was highly toxic and induced significant mortality at the lowest administered dose (5 mg/kg; Fig. 3). Similarly, PM-C20 and PM-C4 showed significant toxicity in zebrafish embryos, causing more than 50% of mortality at concentrations of 10 and 20 mg/kg, respectively, and 100% mortality at 50 mg/kg. Both PM-C4 and PM-C20 were therefore excluded from subsequent analysis. In contrast, PM-C7, PM-C11 and PM-C12 showed low levels of toxicity, with mortality not exceeding 16% at the highest dose tested (50 mg/kg). Unloaded PMs by themselves had no toxic effects and did not induce mortality in injected zebrafish embryos (Fig. S2).

**Fig. 4.**
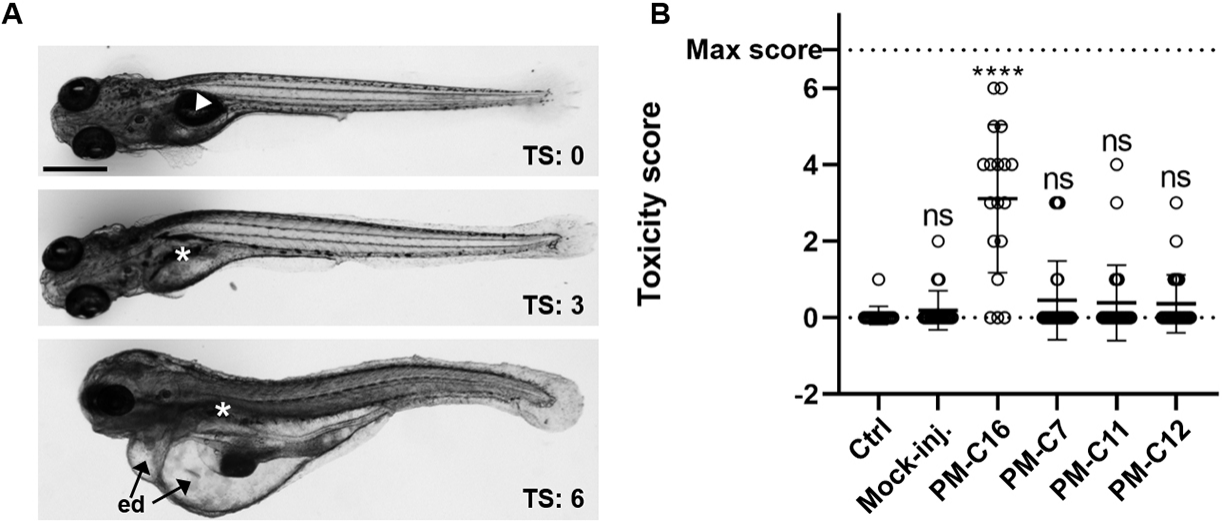
**Evaluation of the *in vivo* toxicity of PM-formulated nNF derivatives in zebrafish embryos by toxicity score.** Zebrafish embryos were injected with high doses (50 mg/kg) of PM-formulated C7, C11 and C12, or 5 mg/kg of PM-C16, and the toxicity score (TS) was determined at 4 dpt in individual embryos based on morphological and physiological indicators of toxicity, as specified in Table 1. (A) Representative images of embryos with different toxicity scores displaying normally inflated (arrowhead) or lack of inflated swim bladder (*), severe edemas (ed) and abnormally curved body shape (bottom image). Scale bar: 500 μm. (B) Toxicity scores in embryos injected with PM-formulated compounds. Non-injected (ctrl) and mock (PBS)-injected embryos were used as negative toxicity controls, and embryos injected with PM-C16 as a positive toxicity control. Each symbol represents the toxicity score of an individual larvae and the mean for each group is shown as a horizontal line with error bars denoting the s.d. Ctrl, *n*=18; Mock-inj., *n*=21; C16, *n*=18; C7, *n*=31; C11, *n*=26; C12, *n*=25); ***P<0.001 compared with the non-injected control (ctrl); ns, not significant (non-parametric Kruskal–Wallis H test followed by post-hoc analysis using Dunn's multiple comparisons test).

To determine whether the selected compounds PM-C7, PM-C11 and PM-C12 could induce sublethal toxicity in embryos, we set up a sensitive toxicity scoring system based on well-established physiological and behavioral toxicity indicators (Table 1). These features are readily observable in embryos and include malformed or curved body shape, lack of swim bladder inflation, slow or absent blood flow and formation of edemas (Fig. 4A). When tested at their highest dose (50 mg/kg), the three selected compounds did not cause significantly higher sublethal toxic effects compared to the non-injected or PBS-injected control embryos (Fig. 4B). Compound-treated groups displayed a slightly higher mean toxicity score than the mock-injected control, which was due to the higher frequency of embryos with a toxicity score of 1 lacking an inflated swim bladder. A few cases of embryos with a toxicity score of 2 to 4, mainly characterized by a lack of an inflated swim bladder, deficient equilibrium and reduced escape response, were also observed.

**Table 1.**
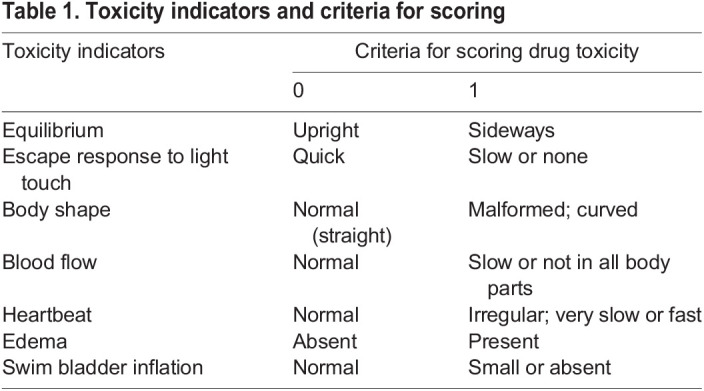
Toxicity indicators and criteria for scoring

In contrast, PM-C16, included as a positive toxicity control, showed a significantly higher toxicity score than the PBS-injected control, at a 10× lower dose (5 mg/kg). In conclusion, PM-C7, PM-C11 and PM-C12 proved to be non-toxic *in vivo*, whereas PM-C4 and PM-C20 were excluded from further analysis due to their significant toxicity.

### Evaluation of three experimental methods to determine the bacterial burden in the zebrafish TB model

**Fig. 5.**
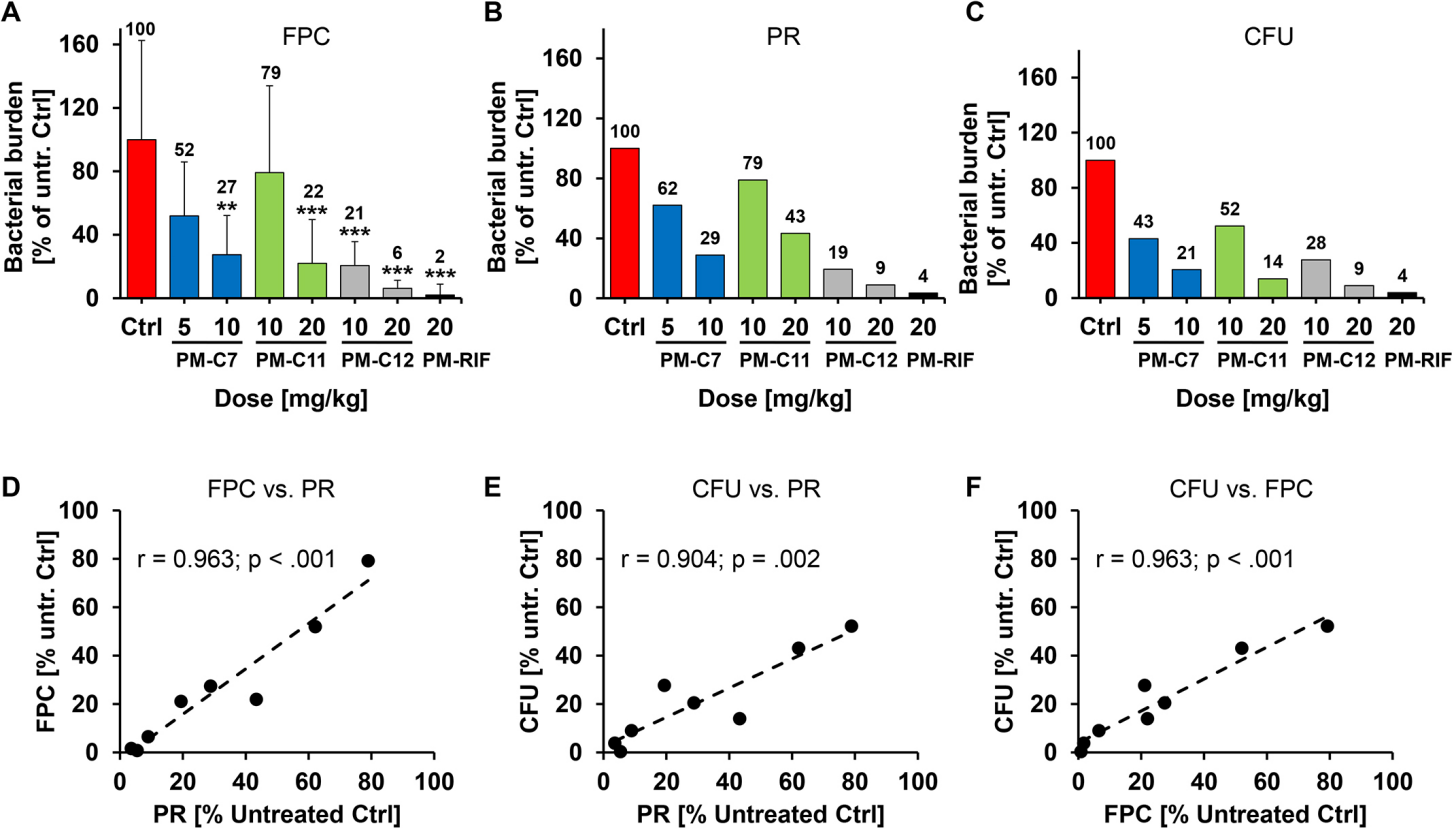
**Quantification of bacterial burden in Mm-infected zebrafish embryos after treatment with PM-formulated nNF derivatives by three different methods.** Mm-dsRed-infected zebrafish embryos were treated with the indicated doses of the PM-formulated nNF derivates C7, C11, C12 or PM-formulated RIF by intravenous injection, and bacterial burden was quantified at 4 dpt by three different assays. (A) FPC: untreated or treated embryos were imaged and the total bacterial fluorescence intensity was determined by FPC for individual embryos. Data are mean±s.d. of data normalized to the untreated control (ctrl) pooled from at least three independent experiments (Ctrl, *n*=71; PM-C7 5 mg/kg, *n*=13, and 10 mg/kg, *n*=15; PM-C11 10 mg/kg, *n*=15, and 20 mg/kg, *n*=18; PM-C12 10 mg/kg, *n*=45, and 20 mg/kg, *n*=49; and PM-RIF 20 mg/ml, *n*=51). **P<0.01, ***P<0.001 (non-parametric Kruskal–Wallis H test followed by post-hoc analysis using Dunn's multiple comparisons test). (B) Plate reader (PR) assay: embryos in each treatment group or the untreated control were pooled and homogenized, and fluorescence intensity was measured using a plate reader. (C) CFU count: bacterial burden was enumerated by plating serial dilutions of the pooled homogenate on 7H10 plates. Results in B and C are shown as a percentage relative to the untreated control and represent data from one experiment. The mean values of each group in A-C are displayed above the columns. (D-F) Correlation between the normalized bacterial burden for all four compounds obtained by the three methods. Linear regression plots comparing FPC with the PR assay (D), CFU with the PR assay (E) and CFU with the FPC assay (F). Pearson's coefficient of correlation (r) with *P*-value are denoted in the individual figures.

We initially compared three different methods for evaluating the ability of PM-formulated compounds to reduce the bacterial burden in Mm*-*infected embryos. Using the three methods in sequence in the same experiment allowed us to directly compare them to each other. First, bacterial burden in individual Mm-dsRed infected embryos was quantified by fluorescence pixel count (FPC) based on the total bacteria-associated fluorescence intensity. Then, embryos within the treatment groups were pooled and homogenized, and the total level of bacterial fluorescence was measured using a plate reader. Finally, the bacterial burden was enumerated by classic CFU plating of the homogenized embryos. Overall, the three methods produced comparable results (Fig. 5A-C), which was reflected in the high degree of correlation observed between them (Fig. 5D-F). Levels of bacterial burden obtained by CFU assay were generally lower than the two other methods, and all three compounds showed a clear dose-dependent efficacy in reducing the number of intracellular bacteria.

### *In vivo* efficacy of PM-formulated nNF derivatives in the zebrafish TB model

**Fig. 6.**
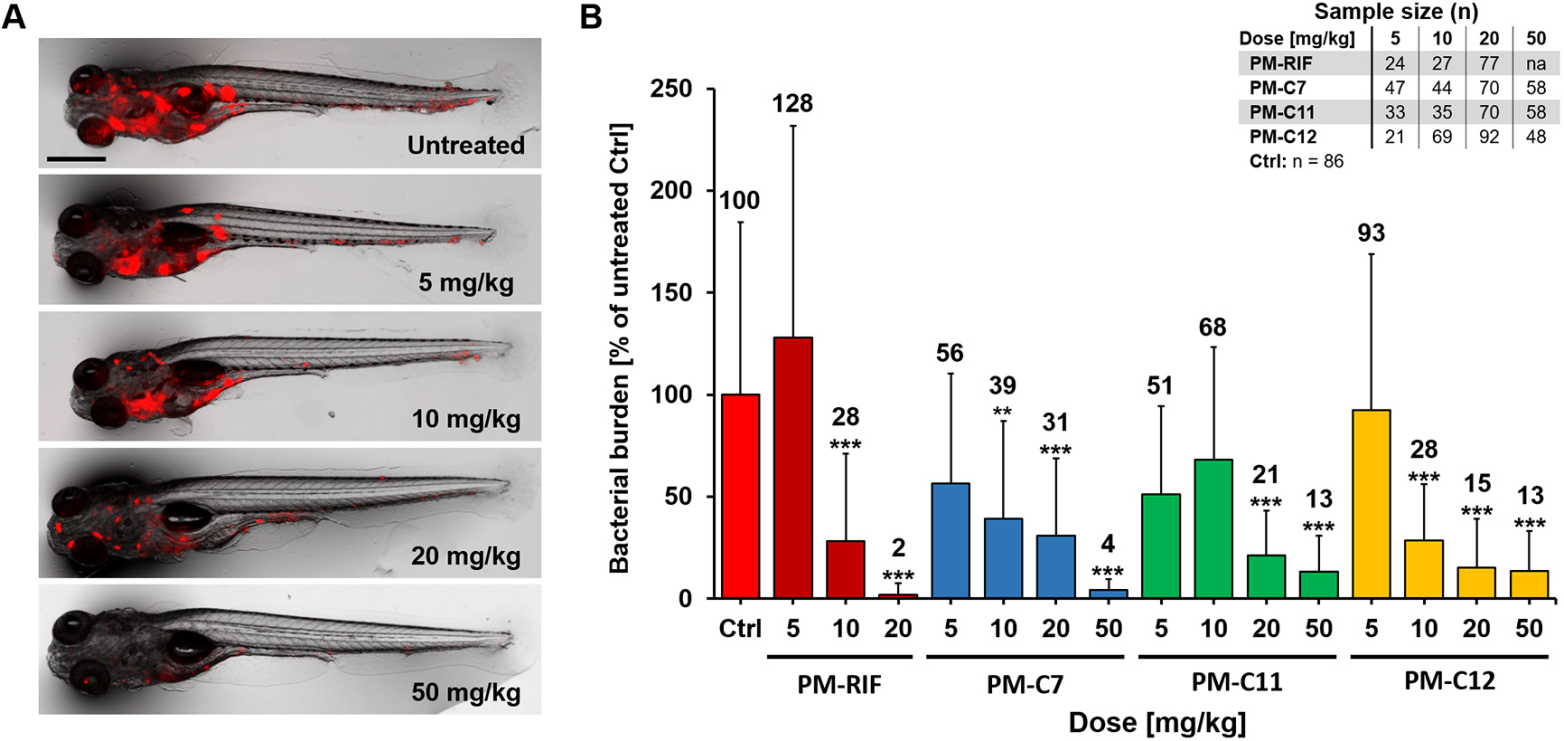
**Evaluation of the *in vivo* efficacy of PM-formulated nNF derivatives in the zebrafish TB model.** Zebrafish embryos were infected with Mm-dsRed and treated with different doses of the PM-formulated nNF derivates C7, C11, C12 or PM-formulated RIF by intravenous injection. (A) Representative images of Mm-infected embryos treated with different doses of PM-C7 at 4 dpt. Scale bar: 500 μm. (B) Quantification of bacterial burden by FPC at 4 dpt in embryos treated with the indicated doses of the PM-formulated compounds or RIF. FPC values of individual embryos were normalized to the untreated control (ctrl) group and results are presented as mean±s.d. Results are pooled from at least three independent experiments. The mean values of each group are displayed above the columns. na, not applicable. **P<0.01, ***P<0.001 (non-parametric Kruskal–Wallis H test followed by post-hoc analysis using Dunn's multiple comparisons test).

Given the excellent concordance between the three methods, we decided to use FPC to evaluate the efficacy of PM-formulated C7, C11, C12 and RIF at a broader range of doses (Fig. 6). All three nNF compounds reduced bacterial burden in infected fish in a dose-dependent manner, consistently showing a significant reduction of bacterial levels at doses of 20 and 50 mg/kg. Compared to treatment with PM-RIF, which was highly effective at a dose of 20 mg/kg, the three tested nNF compounds achieved comparable results at 50 mg/kg, the highest dose tested. Consistent with previous reports by our group (Fenaroli et al., 2014; Trousil et al., 2019), formulation in nanoparticles slightly improved the *in vivo* efficacy of RIF compared to its free form, as measured by bacterial burden in infected zebrafish embryos (data not shown). However, it is unknown whether this is also the case for nNF compounds as it was not possible to test their free form in the zebrafish TB model without the use of high concentrations of toxic solvents.

**Fig. 7.**
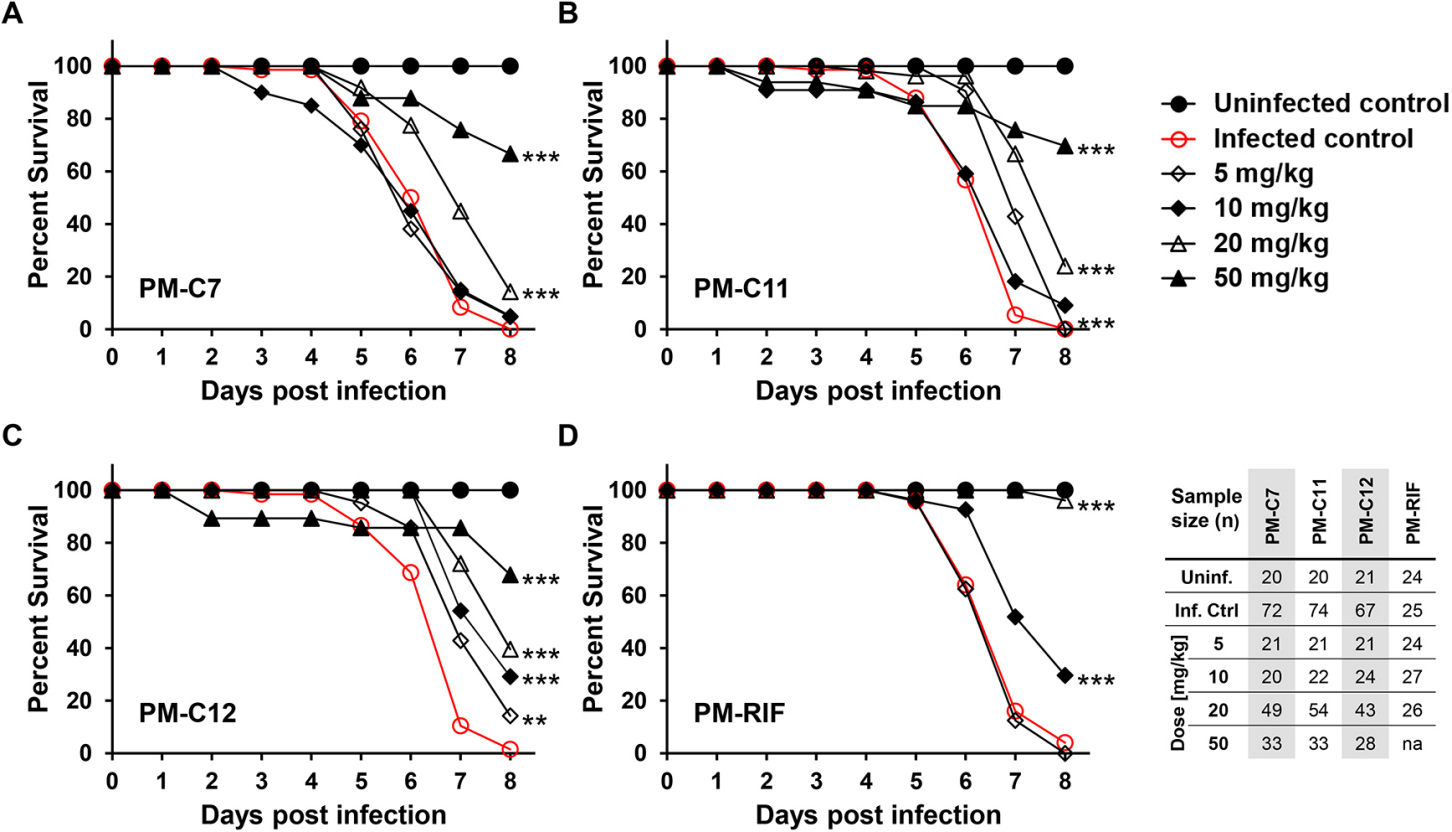
***In vivo* efficacy of PM-formulated nNF derivatives against Mm infection in zebrafish embryos assessed by survival analysis.** Mm-dsRed-infected zebrafish embryos were treated 1 day post infection with the indicated doses of the PM-formulated nNF derivates C7 (A), C11 (B), C12 (C) (5-50 mg/kg) or PM-formulated RIF (D) (5-20 mg/kg) by intravenous injection, and survival was recorded daily. Uninfected untreated embryos were used as a negative control (ctrl) and Mm-infected untreated embryos as an infection control group. Embryos used in this assay are the same as in the FPC assay (Fig. 6). Results are pooled from at least three independent experiments. na, not applicable. **P<0.01, ***P<0.001 (log-rank test of the Kaplan–Meier estimate of survival).

In a final step, we analyzed the ability of the PM-formulated compounds to improve survival in Mm-infected embryos. All compounds significantly improved survival (66%-70%) at 8 days post infection at the highest dose (50 mg/kg) (Fig. 7). In contrast, nearly 100% of untreated embryos succumbed to the infection at the endpoint of the survival assay. However, none of the three compounds showed efficacy comparable to PM-formulated RIF, which achieved more than 95% survival at a dose of 20 mg/kg. Unloaded PMs by themselves had no therapeutic effect and did neither reduce bacterial burden nor improve survival in infected embryos (Fig. S3).

In summary, of the initial six selected nNF-derivatives, three compounds (C4, C16 and C20) were excluded from the study due to their toxicity *in vivo*. The remaining three derivatives (C7, C11 and C12) showed minimal *in vivo* toxicity, effectively reduced bacterial burden and improved survival in Mm-infected zebrafish embryos.

## DISCUSSION

In the treatment of TB, the need for new antibiotics can only be described as urgent (Bandodkar et al., 2020). Phenotypic HTS of large chemical libraries using Mtb or surrogate mycobacteria provide a powerful approach for the discovery of new compounds with anti-TB activity. Those compounds may target new molecular mechanisms in the pathogen, which is especially important for the development of new drugs for the treatment of drug-resistant TB (Wellington and Hung, 2018). On the downside, this approach tends to be biased towards identifying a high proportion of hydrophobic and less water-soluble compounds, which are often difficult to evaluate in the subsequent drug discovery process (Di et al., 2012; Keseru and Makara, 2009; Lipinski, 2000). Encapsulation of hydrophobic molecules in polymer-based nanoparticles offers a powerful solution that is well suited for solubilizing even highly hydrophobic drugs without the use of potentially toxic excipients. In addition, encapsulation of drugs in nanoparticles has been found to reduce drug-related toxicity and improve pharmacokinetic characteristics and bioavailability (Kirtane et al., 2021; Melariri et al., 2015; Vibe et al., 2016). There exists a plethora of nanoparticles for drug delivery, ranging from inorganic to lipid-based and polymeric nanoparticles, which can display a wide range of characteristics and functionalities (Griffiths et al., 2010; Mitchell et al., 2021). Nanoparticles are increasingly being explored as a substitute for conventional pharmaceutical methods for enhancing drug solubility, such as dry or wet milling, the use of surfactants or solid dispersion preparation. Both lipid-based liposomes and polymer-based nanoparticles, such as solid poly-lactic-co-glycolic acid nanoparticles and PMs, have been found to be suitable for the delivery of anti-TB drugs (Grotz et al., 2018). In this study, we have formulated a set of poorly water-soluble nNF compounds with potent anti-TB activity in PMs in order to enhance their solubility, and evaluated their *in vivo* toxicity and anti-mycobacterial efficacy in the zebrafish embryo model. nNFs are aromatic molecules characterized by a nitro group attached to an aromatic ring, a chemical structure they have in common with clinically approved antibiotics, such as nitrofurans like nifurtimox and the new-generation TB drugs delamanid and pretomanid (Rice et al., 2021). These antibiotics are prodrugs that require bioactivation by specific microbial nitroreductases, leading to the release of the highly reactive radical nitric oxide and subsequent damage of microbial DNA, lipids and proteins (Gurumurthy et al., 2012; Hall et al., 2011; Patterson and Fairlamb, 2019; Singh et al., 2008). Whether the activity of nNF compounds against Mtb is based on a similar mode of action is currently being investigated by our group.

It is increasingly recognized that the zebrafish embryo model is a valuable tool for evaluating both the *in vivo* toxicity and efficacy of new drug candidates, thereby bridging the gap between *in vitro* assays and laborious mammalian *in vivo* studies in the drug discovery process (MacRae and Peterson, 2015). Low space requirements and costs for husbandry, their small size, high fecundity and rapid development makes the zebrafish a very attractive model for large-scale studies on drug effects, i.e. *in vivo* toxicity and activity. In such studies, the drugs to be tested have been almost invariably administered to the embryos in the fish water by bath immersion. Although technically easy and convenient, this way of drug delivery is associated with major limitations, especially when studying poorly water-soluble drugs (Cassar et al., 2020). First, it is not possible to deliver defined doses of drugs to the embryo as the degree and route of drug absorption (e.g. via skin, gills or gastro-intestinal tract) is mostly unknown. Second, compounds with low water solubility may be difficult to test at all, or be tested at concentrations high enough to achieve therapeutic concentrations in the embryo. Ordas et al. (2015) have addressed this problem by quantifying the amount of the adsorbed anti-TB drugs in the yolk of bath immersion-treated embryos by mass spectrometry and indirectly relating that value to the observed therapeutic activity in the zebrafish TB model. They found that the degree of uptake was positively correlated to the lipophilicity of the drugs, which also affected the efficacy of the drugs in the zebrafish embryo TB model.

In contrast, in our study, we directly administered candidate drugs to the zebrafish embryos by intravenous injection after encapsulation in nanoparticles, thereby avoiding the above-mentioned issues associated with administration by bath immersion. This approach enabled us to administer precise and reproducible doses of lipophilic compounds with low water solubility, which otherwise would have been difficult to test in the zebrafish larvae. Although in principle possible, the administration of nanoparticle-formulated drugs by bath immersion would face the same issues, i.e. unclear route and degree of drug uptake in the embryos, thus making such experiments generally difficult to interpret. Additionally, drug administration by intravenous injection requires only minimal amounts of compounds, which is especially advantageous when these are used directly from chemical libraries and are only available in limited amounts. Specifically, only a few milligrams are needed to test compounds in the zebrafish embryo, and the result can facilitate the decision to resynthesize a specific compound in larger amounts for testing in mammalian *in vivo* models.

The zebrafish embryo is one of the most sensitive and rapid monitors of drug toxicity, and is widely used for studying adverse drug effects (Cassar et al., 2020). It provides valuable and rapidly obtainable information about the *in vivo* toxicity of compounds, and can be used to screen for, and subsequently discard, toxic compounds early in the drug discovery process. However, in many studies, drug toxicity is still tested exclusively in *in vitro* cell culture systems, which does not allow a reliable extrapolation of drug toxicity to the *in vivo* situation. Our data here provide a clear example of this problem: although the nNF-compound C4 showed minimal toxicity in the cell-based *in vitro* assay, it was highly toxic in the zebrafish embryo, as seen in the mortality assay.

In addition to the mortality assay, which allowed us to identify two of the tested compounds (C4 and C20) as being significantly toxic *in vivo*, we have further defined a simplified toxicity score system in the zebrafish embryo based on previous studies (Palha et al., 2013; Vibe et al., 2016). Using this sensitive toxicity scoring system, we could confirm the low *in vivo* toxicity of the compounds C7, C11 and C12 at the maximum concentration tested (50 mg/kg). Our toxicity score system is uncomplicated and can be further extended and refined, depending on the purposes of the screening, by including additional toxicity indicators. Numerous downstream assays have been developed in the zebrafish embryo for studying tissue-specific toxic effects and mechanisms, including cardiovascular, neuro- and behavioral toxicity and genotoxicity (Cassar et al., 2020; Chakravarthy et al., 2014; Kosmehl et al., 2006). For example, the neurotoxicity of a broad range of drugs has been assessed by studying their effects on the locomotor activity of zebrafish larvae in repeated dark/light cycles (Basnet et al., 2017, 2019). Cardiotoxicity can readily be evaluated as drug-induced bradycardia in the zebrafish embryo was shown to correlate well with QT prolongation in humans (Milan et al., 2003). Importantly, findings from toxicity studies in the zebrafish embryo have been shown to generally correlate well with those obtained in rodent models, such as mice and rats (Ali et al., 2011; Ducharme et al., 2015; Vorhees et al., 2021).

Of relevance here, the zebrafish embryo is an especially well-established model for studying TB (Cronan and Tobin, 2014). Its success has spurred efforts by several research groups to use this model system for the evaluation of the *in vivo* efficacy of new anti-TB drugs. Both clinically approved drugs, such as rifampicin, isoniazid and ethambutol, as well as new drug candidates against TB have been tested in the zebrafish embryo, in part by the use of automated injection and imaging platforms (Dalton et al., 2017; Makarov et al., 2014; Ordas et al., 2015; Takaki et al., 2012). The therapeutic efficacy of anti-TB drugs in the zebrafish TB model is usually scored either by quantifying the survival of Mm-infected embryos or by assessing bacterial burden by the fluorescent pixel count method (FPC), exploiting the transparent nature of the zebrafish in conjunction with fluorescent Mm strains. It is generally accepted that FPC is a good measure of the bacterial burden in Mm-infected zebrafish (Stirling et al., 2020). However, this approach has rarely been directly compared to other methods, in particular CFU enumeration, which is commonly considered to be the gold standard for quantifying bacterial burden in mammalian models of TB (Gumbo et al., 2015).

CFU plating of homogenized whole zebrafish embryos has proven to be quite troublesome because of the high incidence of microbial contamination, most likely due to the presence of commensal bacteria in the gut of the zebrafish larvae and microbial organisms in the surrounding water (Bates et al., 2006; Rawls et al., 2004). Here, we succeeded in establishing a quick and reliable protocol for a quantitative CFU assay in Mm-infected zebrafish embryos that successfully addresses this issue. The most critical steps in our protocol are (1) the incubation of the homogenate with 1% NaOH for 10 min, which effectively eliminates bacterial contamination without affecting Mm viability, (2) the supplementation of the CFU-agar plates with amphotericin B to prevent fungal growth and kanamycin taking advantage of the reporter plasmids carrying a kanamycin resistance cassette as selective antimicrobials, and (3) the dissociation of bacterial aggregates by passaging the homogenate through a 27-gauge needle. When we directly compared the estimation of bacterial burden by CFU plating versus two fluorescence-based methods (FPC and plate reader), we found excellent concordance in the inhibitory activity of the tested drugs between these three methods. In contrast to CFU plating, which detects viable bacteria, fluorescence-based assays may not distinguish between dead and viable bacteria as fluorescent proteins, such as dsRed, can remain stable for several hours in bacteria, even after they are killed (Blokpoel et al., 2003, and our own observation). In contrast, CFU plating might not detect mycobacterial subpopulations, which are viable but grow poorly on solid agar medium (de Knegt et al., 2017; Hu et al., 2015). These two observations may explain the generally higher reduction in bacterial burden seen after drug treatment observed in CFUs compared to FPC or the plate reader assay. Therefore, using both CFU plating and FPC together could represent an ideal experimental combination to improve the accuracy of drug efficacy evaluation in the zebrafish TB embryo model by counterbalancing the drawbacks of the two assays. However, FPC is, in contrast to the classic CFU assay, a non-invasive method that allows one to conduct other assays, such as survival assays in parallel, and therefore often remains the preferred method.

In our study, we have combined several complementary assays in the zebrafish TB model to identify the best compound candidates from a previous drug whole-cell screening in terms of their anti-mycobacterial potency *in vivo*. The nNF compounds C7, C11 and C12 significantly reduced the bacterial burden in infected zebrafish, and improved their survival with minimal toxic effects. These results pinpoint the aforementioned compounds as the most promising candidates for further development and *in vivo* testing in the conventional mouse TB model.

In conclusion, the zebrafish embryo can fill a void in the preclinical evaluation of new TB drug candidates by bridging the gap between *in vitro* studies in bacterial or mammalian cell cultures and time- and cost-intensive *in vivo* studies in mammalian TB models. In a time frame of less than 2 weeks, the zebrafish embryo model can provide valuable information about both the *in vivo* toxicity and efficacy of anti-TB candidate drugs, and thus facilitate the identification of the most promising candidate drugs for further evaluation. The use of nanoparticle technology enables the evaluation of even poorly water-soluble drugs *in vivo*, which otherwise would be difficult to study. Last, but not least, the use of the zebrafish embryo satisfies the desirable principles of reduce, replace and refine (3Rs) for animal studies (NC3Rs, 2021). *In vivo* studies in the zebrafish embryo can efficiently eliminate compounds with significant *in vivo* toxicity and provide important information about the toxic or therapeutic dose range of anti-TB compounds. This, in turn, can contribute to the refinement of experiments in higher vertebrates, such as mice and rats, and reduce the number of animals needed at a later stage in the drug discovery process.

## MATERIALS AND METHODS

### Anti-TB candidate compounds

nNF derivatives C4, C7, C11, C12, C16 and C20 were selected from a set of 15 nNFs derivatives that were identified as being active against Mtb in a phenotypic HTS campaign at the Institute Pasteur. The selection was made according to their high *in vitro* activity against Mtb (MIC_90_<8 µg/ml), and their low (C4, C7, C11 and C12; CC_50_>65 µg/ml) or intermediate (C20; CC_50_=9 µg/ml) cytotoxicity in VERO cells (data not published). This resulted in a selectivity index (SI), as calculated by the ratio of the CC_50_ to MIC_90_, of higher than 12.5 for all compounds. The SI measures the window between cytotoxicity and anti-Mtb activity, and is commonly used to evaluate the safety of new compounds for further development after initial screenings (Manjunatha and Smith, 2015; Orme, 2001). The highly cytotoxic compound C16 (CC_50_=0.3 µg/ml), also known as R7000 (Quillardet et al., 2000), was included in the study and used as a toxicity reference. The water solubility of all the nNF derivatives used in this study was lower than 12 µg/ml.

### Preparation of PM drug formulation

Drug-loaded PMs were prepared with the amphiphilic block co-polymer pGlu(OBn)-block-pSar in combination with the appropriate compound by dual asymmetric centrifugation, as described by Fenaroli et al. (2018). Briefly, the amphiphilic block co-polymer was mixed with the different compounds at a weight ratio specified in Table S2, and centrifugation was performed for 30 min at 3500 rpm with a dual asymmetric centrifuge (SpeedMixer DAC 150.1 CM, Hauschild). The resulting slightly milky solution containing the drug-loaded PM was collected and the size of the PM was measured in 10 mM NaCl by dynamic light scattering, and the surface charge by zeta-potential analysis using a Zetasizer (Malvern Panalytical, UK).

### Bacterial cultures

Mm wild-type M strain and M strain expressing dsRed (Mm-dsRed) were obtained from Dr Monica Hagedorn (Bernhard Nocht Institute for Tropical Medicine, Hamburg, Germany). Mm-dsRed expresses the recombinant fluorescent protein dsRed under the control of the MSP12 promoter and carries a kanamycin resistance cassette. Both Mm strains were grown in Middlebrook 7H9 broth (BD Biosciences, Heidelberg, Germany) supplemented with 10% albumin-dextrose-catalase (ADC) and 0.05% tyloxapol (referred to as complete 7H9 medium) at 31.5°C in the dark without agitation. Kanamycin (25 µg/ml, Sigma-Aldrich) was added to the culture medium of Mm-dsRed.

### Determination of the MIC_90_ of nNF derivatives in *M. marinum*

The MIC_90_ for nNF derivatives was determined using the resazurin microtiter assay adapted from Franzblau et al. (1998). Briefly, twofold serial dilutions of nNF compounds in a concentration range of 16 µg/ml to 0.008 µg/ml were prepared in sterile 96-well plates using Middlebrook 7H9 broth supplemented with 10% Oleic acid-ADC (OADC). The final concentration of DMSO was kept below 1% in each well. For inoculation, 1-3×10^3^ bacteria were added to each well and plates were incubated in sealed plastic bags at 31.5°C for 72 h. Then, resazurin sodium salt (Sigma-Aldrich) was added at a final concentration of 0.003%, and plates were incubated for an additional 48 h. Finally, fluorescence was measured at an excitation wavelength of 535 nm and emission wavelength of 590 nm using a microplate reader (Synergy H1, BioTek). The MIC_90_ was defined as the lowest concentration at which the fluorescence value was equal or lower than 10% of the value for the untreated control.

### Isolation of bone marrow-derived macrophages

Wild-type C57BL/6 mice were purchased from Janvier Labs (Le Genest-Saint-Isle, France). BMDMs were prepared as described previously (Speth et al., 2017). Briefly, bone marrow was flushed from the tibias and femurs of 8-12-week-old mice and red blood cells were removed by incubation in RBC lysis buffer (Sigma-Aldrich). Bone marrow cells were then incubated in RPMI medium/10% fetal calf serum (FCS) supplemented with 30% L929 conditioned medium in non-tissue culture-treated dishes at 37°C in a humidified atmosphere containing 5% CO_2_ for 7 days, with medium changes after 3 and 6 days. Differentiated BMDMs were harvested, frozen and stored at −80°C until use. Throughout the experiments, BMDMs were maintained in RPMI medium/10% FCS supplemented with 10% L929-conditioned medium (complete BMDM medium).

### Anti-mycobacterial efficacy of nNF derivatives in *M. marinum*-infected BMDMs

BMDMs were seeded in 48-well plates at 1.2×10^5^ cells per well in complete BMDM medium and incubated overnight at 32.5°C in a humidified atmosphere containing 5% CO_2_. Mid-log phase Mm cultures were washed three times with PBS/0.05% tyloxapol, sonicated in a bath sonicator for 10 min and passed ten times through a 27 G needle to disrupt bacterial aggregates and to produce a single cell suspension. The bacterial suspension was then diluted in RPMI/10% FCS and used to infect BMDM cultures at a multiplicity of infection (MOI) of 0.1 for 3 h at 32.5°C, which is within the optimal growth temperature range of Mm and is well tolerated by mouse macrophages (Carlsson et al., 2010; Ramakrishnan and Falkow, 1994; Stamm et al., 2003). BMDM cultures were washed three times with prewarmed PBS and incubated in complete BMDM medium containing 100 µg/ml amikacin for 1 h to kill remaining extracellular bacteria. After one wash with prewarmed PBS, infected BMDMs were incubated in complete BMDM medium for an additional 24 h at 32.5°C. Then, nNF derivatives were added to the infected BMDMs at concentrations in the range of 0.125× to 32× their respective MIC_90_, and BMDM cultures were incubated for 3 days at 32.5°C. The final concentration of DMSO was kept at 1% for all groups, and BMDMs incubated with 1% DMSO in BMDM medium were used as a negative control. After 72 h of nNF treatment, BMDMs were washed with prewarmed PBS and lysed in 0.005% SDS. Viable intracellular bacteria were quantified by performing serial dilutions of the lysates and plating on Middlebrook 7H10 agar plates supplemented with 10% OADC. Plates were incubated at 31.5°C up to 10 days before CFUs were counted.

### Zebrafish embryo husbandry

Wild-type zebrafish embryos were obtained from the zebrafish facility at the Faculty of Medicine, University of Oslo, Norway, and at the Norwegian University of Life Sciences, Oslo, Norway. Zebrafish embryos were kept at 28.5°C in embryo water supplemented with 0.003% phenylthiourea (Sigma-Aldrich) to inhibit melanization, and water was changed daily for the duration of the experiment. Experiments using zebrafish embryos and larvae were conducted in agreement with the provisions enforced by the national animal research authority at the Norwegian Food Safety Authority (Mattilsynet).

### Microinjection of the zebrafish embryo in the posterior caudal vein

Microinjections in the PCV of zebrafish embryos were performed as described previously (Fenaroli et al., 2014). Briefly, borosilicate microneedles (GC100T-10, Harvard Instruments) were prepared using a pipet puller (P-97, Sutter Instruments) and backfilled with a solution containing Mm for infection or PM-formulated nNF derivatives for treatment. Zebrafish embryos were sedated by immersion in embryo water supplemented with 0.02% tricaine (Finquel) and placed on a plate containing 2% agarose. Injections were conducted under a stereomicroscope (Leica DFC365FX) using a micromanipulator (Narishige MN-153) and a microinjector pump (Eppendorf Femtojet Express pump). Drug doses (mg/kg) were calculated based on an assumed weight of ∼1 mg for zebrafish embryos at 2-3 days post fertilization (dpf) (Fenaroli et al., 2014; Hu et al., 2000; Vibe et al., 2016).

### *In vivo* toxicity assays in zebrafish larvae

Zebrafish embryos received different doses of PM-formulated nNF derivatives by PCV microinjection at 2 dpf, and mortality was recorded twice a day until 8 days post treatment (dpt). Dead or dying embryos were recorded, removed and euthanized if necessary.

*In vivo* toxicity of PM-formulated nNF derivatives causing low mortality was evaluated in more detail by establishing a toxicity score in individual larvae based on morphological and physiological indicators of toxicity as described previously (Palha et al., 2013; Vibe et al., 2016). Indicators of toxicity and their criteria for scoring drug toxicity are summarized in Table 1. For this screening, zebrafish embryos were microinjected at 2 dpf with the highest dose (50 mg/kg) of PM-formulated nNF derivatives or PBS as a negative control, and were analyzed at day 4 post treatment. For each indicator, a value of 1 was assigned if signs of toxicity were present, and a value of 0 if absent. The toxicity score for individual larvae was then calculated as the sum of the values assigned to the toxicity indicators with a maximal possible value of 7.

### *In vivo* efficacy assays in zebrafish larvae

Zebrafish embryos at 2 dpf were infected by injecting ∼500 CFU (5 nl of 10^8^ CFU/ml) of Mm-dsRed in the PCV. The embryos were allowed to recover for 24 h before receiving different treatment formulations by PCV injection. Different doses were administered by adjusting the injected volume or by different dilutions of the treatment formulation.

#### Zebrafish survival study

Zebrafish embryos were checked 1 h after the treatment injection, and only alive and healthy fish were included in the experiment. Survival of embryos was recorded daily for all groups at the same time, and dead embryos or embryos at the humane endpoint were removed and euthanized if necessary. The humane endpoint was defined by the presence of one or more of the following criteria: a heartbeat below 50 beats per minute, the presence of more than one edema and a severely malformed body shape.

#### Determination of bacterial burden by fluorescent pixel count

Mm-infected zebrafish embryos were imaged at day 4 post treatment using a Leica DFC365FX stereomicroscope, with a 1.0× planapo lens obtaining images of the whole fish (30× magnification). The FPC for each individual embryo was quantified using a customized macro in ImageJ (National Institutes of Health), which is accessible at www.github.com/wohlmann/ZF_FPC. Briefly, background noise and autofluorescence were removed by applying a lower threshold filter, and the total fluorescence of Mm-dsRed in individual embryos was calculated as the sum of pixel fluorescence intensity values.

#### Determination of bacterial burden by plate reader assay and colony forming unit enumeration

At 4 dpt, zebrafish embryos were euthanized by an overdose with tricaine (3 mg/ml) and carefully rinsed in PBS. Between 10 and 15 larvae in a volume of 500 µl were transferred into a lysing matrix tube containing 1.4 mm ceramic spheres (MP Biomedical). Homogenization was performed using a FastPrep FP120 cell disrupter (Therma; settings: speed=4, time=10 s) and homogenates were placed on ice for 5 min.

For the plate reader assay, 100 µl of the homogenate was transferred to a black 96-well plate and fluorescence was measured at an absorption wavelength of 550 nm and emission wavelength of 583 nm using a microplate reader (Synergy H1, BioTek).

For CFU plating, the homogenate was transferred to a microcentrifugation tube and passed ten times through a 27 G needle to disrupt mycobacterial clumps. Then, NaOH was added to a final concentration of 1% and incubated at room temperature for 10 min before serial dilutions in PBS were plated on 7H10/10% OADC agar plates containing 10 µg/ml amphotericin B and 25 µg/ml kanamycin A. Plates were incubated at 31.5°C for up to 14 days before CFUs were enumerated.

### Statistics

All statistical analysis was performed using GraphPad Prism 8 (GraphPad Software, San Diego, CA, USA). The normality of data distribution for control and treatment groups was tested using the D'Agostino–Pearson normality test. In the toxicity score and FPC experiments, treatment groups were compared to the control group using the non-parametric Kruskal–Wallis H test followed by post-hoc analysis using Dunn's multiple comparisons test. The effect of treatment on the survival of Mm-infected zebrafish embryos was analyzed using a log-rank test of the Kaplan–Meier estimate of survival.

## Supplementary Material

Supplementary information
