# Supplementary material for: The zebrafish embryo as an in vivo model for screening nanoparticle-formulated lipophilic anti-tuberculosis compounds
Source: Dis Model Mech. 2022 Jan 26;15(1):dmm049147. doi: 10.1242/dmm.049147 (PMC8807572; doi:10.1242/dmm.049147)
Supplement: Supplementary information [file dmm-15-049147-s1.pdf]

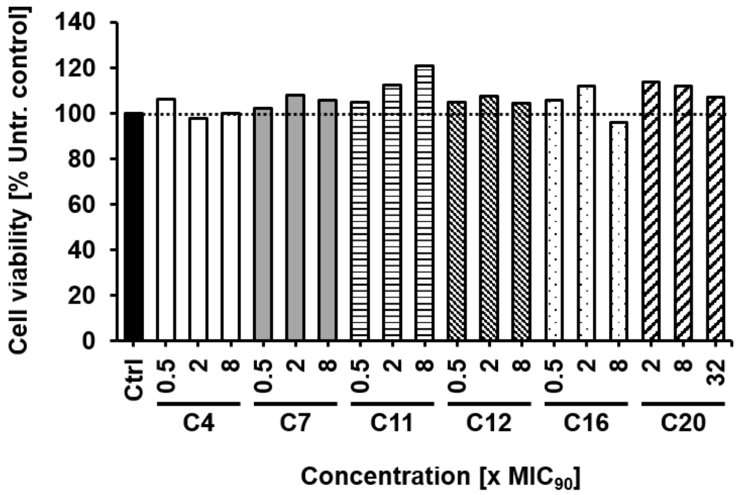

Fig. S1. Cytotoxicity of nNF-derivatives in bone marrow-derived macrophages (BMDM). BMDM cells were treated with nNF-derivatives at concentrations ranging from 0.5 to 32 times their MIC for three days and cell viability was measured using the CellTiter-Glo® cell viability assay (Promega Corporation, Wisconsin, USA) according to the producer's protocol.

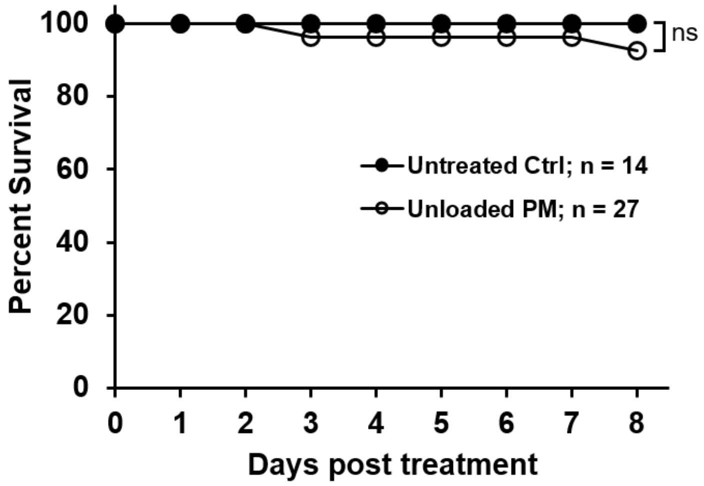

Fig. S2. *In vivo* toxicity of unloaded PM in zebrafish embryos. Zebrafish embryos were treated with unloaded PM at a dose equivalent to 150 mg/kg PM-formulated drug by intravenous injection. Mortality was recorded daily until 8 days post treatment and untreated embryos served as negative control. Data represent one experiment and was repeated twice with similar results; ns: not significant.

Disease Models & Mechanisms • Supplementary information

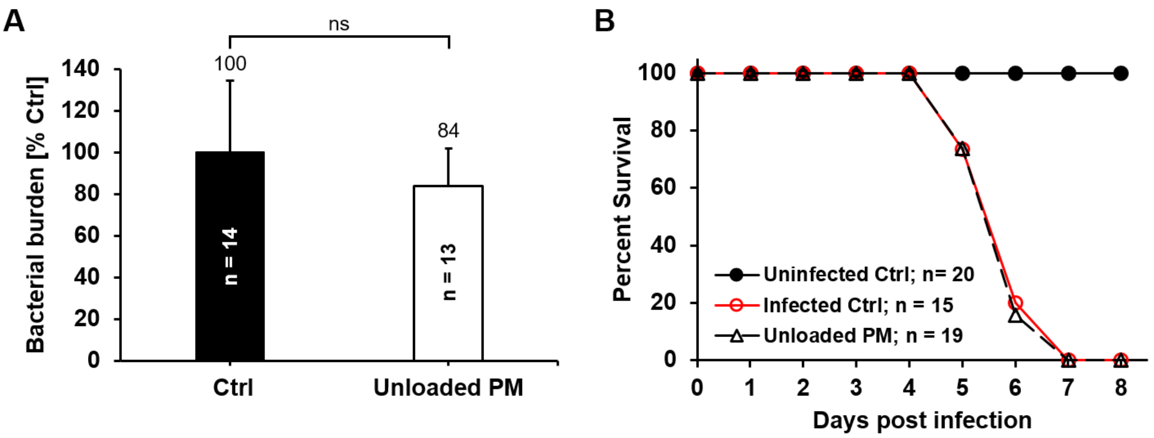

Fig. S3. Effect of unloaded PM on bacterial burden and survival in zebrafish embryos infected with *M. marinum*. Zebrafish embryos were infected with *M. marinum*-dsRed and treated 1 day post infection with unloaded PM at a dose equivalent to 75 mg/kg PM-formulated drug by intravenous injection. (A) Quantification of bacterial burden by FPC at 4 days post treatment in *M. marinum* infected embryos treated with unloaded PM or left untreated. FPC values of individual embryos were normalized to the untreated control group and results are presented as the mean ± SD. The mean values of each group are displayed above the columns. (B) Survival of the zebrafish embryos used in the FPC assay over a period of 8 days post infection. Uninfected, untreated embryos served as negative control. Data in A) and B) represent one experiment and was repeated twice with similar results; ns: not significant.

Disease Models & Mechanisms • Supplementary information

Table S1. *In vitro* activity of nNF-derivatives against extracellular *M. marinum* and intracellular bacteria in bone marrow-derived macrophages (BMDM). The IC_{90} and IC_{99} of nNF-derivatives against intracellular *M. marinum* were calculated from the data of the macrophage infection assay shown in Fig. 2 and compared to the activity against extracellular bacteria by calculating the ratio between IC_{90} and MIC_{90}.

Drug	MIC_{90} µg/ml (µM)	IC_{90} µg/ml (µM)	IC_{99} µg/ml (µM)	IC_{90}/MIC_{90}
C4	0.063 (0.23)	0.05 (0.2)	0.11 (0.4)	0.86
C7	0.016 (0.06)	0.007 (0.03)	0.015 (0.06)	0.44
C11	0.5 (2)	0.31 (1.25)	0.66 (2.62)	0.62
C12	0.25 (0.9)	0.12 (0.45)	0.25 (0.89)	0.56
C16	0.016 (0.07)	0.013 (0.06)	0.03 (0.11)	0.82
C20	0.016 (0.05)	0.4 (1.24)	0.78 (2.45)	24.82
RIF	0.1 (0.12)	0.878 (1.07)	1.97 (2.41)	8.78

Table S2. Physicochemical characterization of polymeric micelles (PM) incorporating nNF-derivates. The surface charge (zetapotential), mean hydrodynamic diameter and polydispersity index (PDI) of nNF derivative-encapsulating PM were determined by Dynamic light scattering (DLS) using a Zetasizer (Malvern Panalytical, United Kingdom).

Name	Cargo	% weight drug/polymer	Surface charge [mV]	Diameter [nm]	PDI
PM-C4	C4	14	-3.3 ± 0.1	130 ± 1	0.15 ± 0.01
PM-C7	C7	20	-3.5 ± 0.2	149 ± 1	0.15 ± 0.02
PM-C11	C11	20	-3.3 ± 0.4	93 ± 2	0.13 ± 0.01
PM-C12	C12	20	-3.3 ± 0.3	89 ± 1	0.11 ± 0.01
PM-C16	C16	20	-2.5 ± 0.1	85 ± 2	0.12 ± 0.02
PM-C20	C20	20	-2.6 ± 0.1	126 ± 1	0.12 ± 0.02
PM-RIF	Rifampicin	10	-2.5 ± 0.4	84 ± 0(.4)	0.14 ± 0.01
